# Developing Life Stories for Nursing Home Residents and Examining the Impact on Residents and Staff

**DOI:** 10.1093/geroni/igaa057.144

**Published:** 2020-12-16

**Authors:** Miriam Rose, Farida Ejaz, Brian Polk

**Affiliations:** Benjamin Rose Institute on Aging, Cleveland, Ohio, United States

## Abstract

A life story program was implemented in 16 nursing homes (NHs) in Ohio with partners including a company specializing in life story work and a gerontological institute. The aim was to evaluate the impact of the life story program on residents and staff. NH sites were selected from an urban/suburban and a rural county using sampling procedures ensuring variation in auspice, quality star ratings and bed size. A longitudinal design was used to conduct in-person interviews with residents at baseline (prior to the life story interview), immediately after the interview, and approximately a month after most life story books were delivered to a NH. Resident eligibility criteria included being age 60 or older, Medicaid-eligible, long-stay and having no to moderate cognitive impairment. Residents’ (n=238) average age was 77 years, 66% were female, and 52% had resided in the NH for 1-5 years. Cognitive scores declined over time, but depressive symptomatology improved significantly. Residents had very high levels of satisfaction with care, enjoyed telling their life stories and would recommend the program; these findings did not change. A pre-post study design was used with staff (n=198), who included nurse aides, nurses, administrators, social workers and activity staff. Their average age was 44 years. Although staff job satisfaction did not change significantly, the vast majority enjoyed learning about residents’ life stories and used them in care planning. The findings demonstrate that life story work may be useful in promoting person-centered care, although further testing is needed with a more generalizable sample.

